# Understanding Ukrainian military chaplains as defenders of the human soul

**DOI:** 10.3389/fsoc.2025.1559023

**Published:** 2025-03-12

**Authors:** Jan Grimell

**Affiliations:** Department of Sociology, Faculty of Social Sciences, Umeå University, Umeå, Sweden

**Keywords:** Ukraine, war, military chaplains, human soul, *Me*, *I*, moral, character

## Abstract

The aim of this article was to explore the stresses of war on the human soul, utilizing empirical research on the experiences and contributions of military chaplains (MCs) in the war in Ukraine. The concept of the human soul was examined through a theoretical framework inspired by Mead's notions of the *I* and the *Me*. The *I* represented the unique, creative, and transcendent aspects of a person, while the *Me* reflected the cultural and social constructs that integrated individuals into broader socio-cultural contexts. This interplay between the *I* and the *Me* formed the basis for understanding the human soul as both transcending culture and deeply embedded within it. The empirical material was derived from a qualitative interview study conducted in 2024 with 12 Ukrainian MCs. Data analysis employed thematic coding using an inductive approach, resulting in the identification of key themes related to the moral, ethical, and character dimensions of military service. An abductive approach was employed in the analysis, which allowed concepts to cross-fertilize the key themes. The findings revealed that war disrupted the social structures, norms, and values that underpin peaceful societies, profoundly impacting the mental health of military personnel. MCs played a crucial role in mitigating these effects by fostering moral coherence, upholding ethical standards of the *Me*, and safeguarding the human *Me* of soldiers in the face of dehumanizing wartime conditions. Their work was deeply rooted in cultural and religious traditions, enabling them to address existential and moral issues that transcended the scope of conventional medical interventions. By offering confidential pastoral care, MCs created spaces for military personnel to process and interpret their experiences, reconnect with their moral and spiritual identities, or Me's, and maintain operational effectiveness. This pastoral, culturally grounded approach complemented—and, in some cases, surpassed—medical models in addressing the complex challenges of existential mental health during war. The article underscored the need for a more holistic understanding of war-related mental health challenges, emphasizing the importance of integrating cultural, moral, and religious/spiritual dimensions into care frameworks.

## Introduction

Military chaplains (hereafter MCs) are generally tasked today with providing pastoral and spiritual care in military settings, conducting religious and spiritual rituals, and fulfilling additional roles, such as teaching ethics and morality or advising military commanders (Bock, 1998; Carey et al., 2016; Koenig et al., 2023; Liuski and Grimell, 2022; Stallinga, 2013). The presence, mission, and purpose of MCs are governed by the socio-cultural traditions, legislation, and military regulations of each country. It is essential to take a nation's culture and traditions into account to understand the role and function of MCs (Grimell, 2024).

The term MCs refers to a modern designation for an ancient phenomenon: the presence of priests and other religious representatives accompanying military personnel and units during times of conflict and war (Carey et al., 2016; Gudmundsson, 2014). This phenomenon is not exclusively tied to contemporary perspectives on MCs, their roles, or their tasks. Historically, religion in Western societies—specifically Catholicism and Protestantism—was deeply intertwined with power, politics, and warfare (Harrison, 2016, 2019). However, secularization has fundamentally reshaped this relationship with power, authority, and state institutions, albeit to varying degrees depending on the specific socio-cultural context within the Western world (Casanova, 1992, 1994).

One thing that has not changed too much, despite the significant transformations many Western societies have undergone over time, is the fact that nations and military personnel continue to wage war and kill each other. The act of killing in war, as well as war itself, may even be considered good (LiVecche, 2021) and just (Holmes, 2005). The framings of war as good and just depend on the point of view that is adopted. A certain war frame may have political/power, legal, ethical/moral, and even theological layers embedded in a particular understanding of both the war and the act of killing. Different points of view exist side by side; it is in the nature of war to have at least two antagonists or more.

This archaic practice of state-organized killing, whether in defense against attacks or as a preemptive measure, remains a shared feature of most societies today (Huntington, 1957; Moskos et al., 2000; Wilson, 2008), as painfully evident in the full-scale war in Ukraine. MCs also hold a well-defined place within the war apparatus in Ukraine (Grimell, 2025a,b) and beyond, and their role can be understood as far more expansive than simply providing pastoral and spiritual care or performing religious and spiritual rites.

The toxic reality and stresses of war have mental health implications for military personnel, which, in the long run, can lead to medical consequences and be understood through a dominant medical perspective (e.g., alcoholism, depression, suicide). However, the root of these symptoms may rather lie in the fact that a human being is a socio-cultural construct with certain values, meanings, and practices—an analytical concept that can be referred to as a socio-cultural *Me* (Mead, 1934/2015).

The brutality of war erodes this *Me*, gradually dissolving the human aspect of a soldier and potentially creating a war animal (Grimell, 2025b). Preventing and countering the erosion of norms, values, and ethics/morals is a key focus of Ukraine MCs, who systematically work to address this issue (Grimell, 2025b). MCs can thus be understood as a distinct group with cultural competence outside traditional military medicine (psychiatry, psychology), working to preserve the existential mental health of military personnel and veterans. They embody an important *healing role* that both broadens and complements an overly narrow medical perspective in a complex situation (Illich, 1977).

### Aim

The aim of this article is to reflect on the stresses of war on the human soul (conceptualized as consisting of a unique *I* and socio-cultural *Me*), through empirical research on the work and contributions of MCs in the war in Ukraine.

Until the specific interview study with Ukrainian MCs conducted in 2024, which this article draws its material from, there was no English peer-reviewed published research on Ukrainian MCs and their lessons learned from the war. Two publications from the study have since been released to outline the results in broader terms (Grimell, 2025a,b). This article, however, delves deeper into the material to explore a specific thematic area concerning the war's impact on the human soul.

The article will continue by briefly presenting military chaplaincy, Ukrainian military chaplaincy, conceptually defining the term human soul, and finally outlining the method, analysis, and discussion.

### A brief overview of military chaplaincy as a practice and research field

Military chaplaincy, both as a practice and a research field, has gained renewed relevance in light of the many different operations that have followed the conflicts and wars of the 21st century (Grimell, 2024). Most armed forces, especially within NATO (Bock, 1998), have military chaplaincy services, but even countries outside NATO, such as Ukraine (Grimell, 2025a) and Russia (Gustafsson Kurki, 2024), have MCs. In some NATO member countries, such as Sweden, the function has existed for almost 500 years (Grimell, 2024).

The presence of MCs in armed forces is supported by legislation, culture, and tradition, which can vary between countries (Grimell, 2024). Often, but not always, the presence of MCs is driven by people's right to practice their faith and religious identities. This also means, though not always, that armed forces may have a multifaith representation of MCs, ranging from religious and spiritual traditions to humanist (secular) perspectives. However, in several armed forces (both within NATO and other examples), MCs can also perform other tasks of a more general nature, such as addressing and educating military personnel on ethical and moral issues.

Research on military chaplaincy has intensified over the past few decades and has been recognized as an important partner in military medicine. This can be linked to the emergence of the concept of moral injury (Shay, 2002, 2003; Litz et al., 2009) and the growing realization among clinicians that moral injury is better addressed by MCs (and clergy) than by clinicians who are not trained in the existential, religious/spiritual approach that such an injury requires (Besterman-Dahan et al., 2012; Bobrow et al., 2013; Litz et al., 2009; Wortmann et al., 2017).

Currently, research on military chaplaincy is often found as a subfield in religious, spiritual, and chaplaincy-focused journals, as well as in medically oriented journals, particularly those in psychiatry and psychology. However, there are also dedicated journals on military chaplaincy, such as the *Australian Army Chaplaincy Journal* (published by the Australian Government, Department of Defense) and *U.S. Army Chaplain Corps Journal* (the official publication of the U.S. Army Chaplain Corps).

### Description of Ukrainian military chaplaincy

After Ukraine declared independence from the Soviet Union, priests and deacons began providing voluntary support to the Ukrainian military. For more than 20 years, pastoral military support relied on the voluntarism of the clergy. As a result, the Military Chaplaincy of the Armed Forces of Ukraine is likely the most recently formalized and professionalized state-led chaplaincy service in the world.^1^

In late 2021, shortly before Russia's full-scale invasion of Ukraine, the Ukrainian Parliament (the Verkhovna Rada) passed a law establishing a military chaplaincy. This law, titled “*On the Military Chaplaincy Service,”* came into force on 1 July 2022. The Military Chaplaincy Service is now an official structure within the Armed Forces, the National Guard, the State Border Guard Service, and other military formations (Grimell, 2025a,b).

Prior to 2022, the presence of clergy in military units and at the front lines consisted of voluntary efforts by priests. On a volunteer basis, these clergy provided pastoral care and support to military personnel to the best of their abilities. Priests often stayed with military units at the front for weeks, months, or even longer periods, moving independently between frontline positions—often on foot—without the centrally regulated armed and experienced assistants (soldiers) that are now standard (Grimell, 2025a,b).

While the Military Chaplaincy Service of the Armed Forces of Ukraine is newly established as a professionalized structure, it includes many highly experienced wartime MCs. Several of these MCs have been active since the onset of the war in 2014, when Russia annexed Crimea, and they played a key role in the development and professionalization of the chaplaincy framework. Some of these MCs are arguably among the most experienced wartime MCs in the world, with nearly a decade of service in war conditions (Grimell, 2025a,b).

The Military Chaplaincy Service is multifaith, reflecting Ukraine's religious diversity. It includes Orthodox, Greek Catholic, Protestant, Jewish, and Muslim chaplains. Religious organizations and communities are responsible for evaluating the suitability, appointing, and providing spiritual formation for MC candidates. The Armed Forces employ these appointed MCs, who undergo military preparatory training based on their backgrounds and experience.

The MCs' four primary responsibilities are:

- Pastoral Care
- Religious and Educational Work
- Social and Charitable Activities
- Advising Commanders on Spiritual and Religious Issues

There are generally two types of MCs in the Armed Forces of Ukraine: brigade-level MCs (typically holding the rank of major) and battalion-level MCs (usually captains). Brigade MCs focus on organizing chaplaincy work within the brigade, taking on a more administrative and coordination-focused role. They work relatively closely with other functions, such as the brigade psychologist, and oversee the activities of battalion MCs.

Battalion MCs operate at a lower tactical level, directly delivering chaplaincy services to soldiers and their units. In situations where there is no assigned battalion MC, the brigade MC steps in to provide pastoral care for that battalion as needed.

MCs are non-combatants and are accompanied by an armed soldier when working near the front lines.

Given the dynamic nature of the war, with new units constantly being formed and the chaplaincy structure still undergoing professionalization, exceptions, adaptations, and improvisations in the deployment and roles of MCs are not uncommon. In cases where, for example, battalion MCs are lacking, brigade MCs can support the battalions by also assuming the role of a battalion MC. If an MC is missing in a heavily combat-engaged battalion, an MC from an entirely different branch of service may be ordered to support the battalion for an extended period. Given the dynamic nature of the war, the formation of new units, the ongoing establishment of the Military Chaplaincy Service in the Armed Forces of Ukraine, and the fact that MCs are injured or killed, one should exercise caution and restraint when making generalizations about MCs in Ukraine.

For further details see Grimell (2025a,b).

### Conceptualizing the human soul

The soul is a deeply rooted concept in culture and human thought. Religious, philosophical, and cultural perspectives offer varied explanations and interpretations of what the soul truly means. Defining the soul is challenging due to its complexity and diverse meanings (Kurkiala, 2019).

The strength of a concept like the soul lies in its status as a deeply ingrained cross-cultural idea that both secular and religious theorists can engage with. The concept of the soul is widely accepted in religious traditions, such as Christianity and Hinduism, as well as in secular fields of study (for instance, Braidotti, 2006; Moss and Prince, 2014; McLaren, 2002; Tick, 2005), including philosophy (Foucault, 1979). A key dividing line often involves the connection to a transcendent dimension, which, from a religious perspective, can encompass existence after death and the immortality of the soul.

In general, the soul can be described as unique to each person, a bodiless reality (Foucault, 1979). The soul is an immaterial, spiritual, or conscious and unique entity believed to reside within every human being (Grimell, 2018). It is often associated with questions of human identity, character, consciousness, self-awareness, and an existence beyond the physical body (Brock and Lettini, 2013; Graham, 2017; Grimell, 2018; Tick, 2005). Regardless of whether one takes a secular or religious view of the soul, it is seen as beyond and more than the physical body (Grimell, 2024).

There is a strong relationship between the soul and the concept of spirit, which can be understood as synonymous but also nuanced depending on usage or distinctions made (Grimell, 2018). Regardless of distinctions, theorists from fields such as social psychology, psychology, pastoral care, and theology tend to describe the soul and spirit as an inner, unique, feeling, integrative, and creative part of a human being (Graham, 2017; Grimell, 2018; James, 1890; Pargament and Sweeney, 2011; Tick, 2005; Tillich, 1952/2014).

This article presents a sociologically and social-psychologically developed version of the soul.

### A mead-inspired conceptualization of the human soul

Mead (1934/2015) described the self as consisting of two parts: the *I* and the *Me*. The *I* represents the active and acting part of the self, existing in the present (Mead, 1934/2015, p. 77). It is the ephemeral, unique, and creative agent of the self. The *Me*, on the other hand, is the social roles or cultural characters reproduced and created through the generalized other, which can be understood as society's influence on the self. The *Me* helps the self understand, interact with, and define situations in light of cultural symbols—particularly language, values, and practices—that emerge from a specific social context (Mead, 1934/2015, p. 209). The *I* and *Me* coexist in a reciprocal relationship, where the uniqueness, spontaneity, and creativity of the *I* are tempered by the *Me*, which exercises social governance over the *I* (Mead, 1934/2015, p. 210). Society's values control the *I* through the *Me*, and every group to which a person belongs creates a distinct *Me*. Humans, therefore, juggle multiple *Mes* that coexist and influence the *I*.

Mead's (1934/2015) version of the *I* was not as fully elaborated as James's earlier version (1890), which also distinguished between the *I* and the *Me*. In James's model, the I represented the aspect of the self that felt, experienced, thought, and was conscious, whereas Mead described the I in terms of creativity, spontaneity, and agency. In both Mead's and James's model, the *I* is the original, genuinely unique part of the self, while the *Me* is culturally constructed. Both parts are deeply interdependent and essential for a person to become human in a social context.

The Mead-inspired concept of the soul developed in this article repurposes the same theoretical framework—the *I* and the *Me*—to illustrate the human soul. The *I* represents the original, creative, and unique aspect of a person, the part that feels, experiences, thinks, and is conscious. The *I* transcends culture—that is, it is greater than, beyond, and prior to culture. However, culture and symbols, represented by the *Me*, are necessary for the *I* to become human. Without the “human” prefix and part of the concept, the *I* would lack orientation points and interpretive keys. The *Me* creates meaningful symbolic interaction and context. The Me has emerged in a cultural and symbolic everyday context that provides it with a certain sense of coherence—SOC, as Antonovsky (1987) describes it—which is connected to comprehensibility (i.e., understanding events in some kind of rational way), manageability (i.e., having the resources to handle a situation), and meaningfulness (i.e., the ability to create emotional meaning and existential motivation in a situation). At the same time, the *Me* is a part of the person that transcends the individual, integrating and embedding the individual in culture, symbols, and relational contexts.

Thus, the human soul transcends culture through the *I* and the individual through the *Me*. This reciprocal relationship is vital but also indicates that the human soul, via the sensitive and unique *I*, can be harmed by events with negative or destructive effects on the *I*, or by a cultural and symbolic *Me* that subjugates or violates the *I* (Grimell, 2023).

## Methods

The empirical material utilized in this article is drawn from a qualitative interview study with Ukrainian MCs conducted in 2024. The study was initiated by the Chief Chaplain of the Swedish Armed Forces and aimed to gather experiences and learn from seasoned wartime MCs. The study was approved by the Swedish Ethical Review Authority (Reference number 2023-05049-01). Although key aspects of the findings have been published previously (Grimell, 2025a,b), nuanced details and certain implications of the material remain unexplored. This article delves deeper into analytical themes that can particularly be related to what has come to be conceptualized as the human soul. By doing so, it aims to reflect on the impact of war on the human soul through the lens of medical sociology.

### Sample and selection

The study included 12 male participants (*N* = 12) recruited via two different points of contact combined with a snowball sampling method (Noy, 2008). Given the participants' ecclesiastical backgrounds and their broad and in-depth experiences of the ongoing war, qualitative saturation was satisfactorily achieved.

### Selection criteria

Selection criteria required participants to have a background as priests or pastors within the Christian church family and to have served as MCs for as long as possible (ideally since the war started in 2014) to provide the most extensive experience possible. Since the study was initiated by the Swedish Armed Forces to learn from MCs in war, there was also an intention for the sample to resemble Swedish MCs as closely as possible—for example, in terms of their background as parish priests, ecclesiastical ordination, and affiliation with the Christian church family (Grimell, 2025a).

For priests within the Christian church family, aspects such as parish life (baptizing, confirming, marrying, burying, pastoral care, etc.), theology, liturgy, rituals, and the ecclesiastical office are more closely connected compared to entirely different religious traditions and communities, such as Jewish or Muslim ones. There are, of course, also differences, yet there is also considerable overlap that can facilitate understanding and applicability.

This intention was achieved, as the 12 participants generally reflected the parish backgrounds of Swedish Lutheran (Protestant) MCs/priests and were distributed within the Christian church family as follows: six Orthodox, four Greek Catholic, and two Protestant.

One challenge was the gender dimension. In Sweden, many MCs are women due to the Lutheran Church's tradition of ordaining both men and women. This was difficult to reflect, as the Orthodox and Greek Catholic traditions in Ukraine do not allow women to serve in ecclesiastical ministries.

### Sample characteristics

To minimize the risk of identifying participants, they are described only at a group level.

In general terms, the majority of the participants were highly experienced MCs in the Ukrainian context. A handful had served as volunteer MCs since the early 2000s, with some serving even longer. Nine participants had been volunteer MCs at the frontline since the war began in 2014. During this period, they served as parish priests and periodically volunteered to support military personnel at the front—spending weeks or months there before returning to their regular duties and then going back to the front again. These volunteer MCs have since been employed by the Armed Forces of Ukraine or integrated into the military chaplaincy structure, reflecting the institutionalization and professionalization of military chaplaincy.

One participant in the study remained a volunteer MC. Two participants had much shorter careers as MCs, having been employed during the professionalization process that began in 2022. Nonetheless, they had served as MCs at the frontline with units engaged in heavy combat over the past 2 years. While their experience was less extensive compared to their senior Ukrainian colleagues, it was significant in comparison to their international peers.

Most participants were aged between 40 and 50, with one just over 50 and another just over 30.

The participants represented a mix of both battalion- and brigade-level MCs, although there was a slight predominance of brigade MCs. Many participants had served in both roles and as volunteer MCs prior to and during the professionalization, reflecting their extensive service. The traditional branches of the Armed Forces—the Army, Navy, and Air Force—were represented, as well as other units not named here due to their unique nature. Of the 12 participants, 11 belonged to or had served in one or more frontline combat units. For further information, see Grimell (2025a,b).

### Interview design

Participants received study information in both Ukrainian and English. The interview questions and informed consent forms were also translated into both languages. Participants were required to return signed consent forms before interviews were conducted.

The interviews were carried out via digital communication platforms. In some cases, participants provided written responses due to the logistical challenges posed by the wartime frontline context. It turned out to be a major challenge to arrange the interviews with frontline MCs belonging to units engaged in combat (see Grimell, 2025a). Eight participants were interviewed, while four submitted written responses.

A semi-structured interview guide was developed to cover topics relevant to the study, ranging from background information, tasks, roles, moral, spiritual, and existential challenges in war, to aspects of maintaining the ability to conduct military chaplaincy during wartime (see Appendix 1). The guide also allowed for unplanned follow-up questions to clarify or expand on responses.

### Language and interpretation

Language barriers arose during the interviews, as not all participants spoke English and the researcher did not speak Ukrainian. Therefore, a Ukrainian-Swedish interpreter from the Swedish Armed Forces assisted in seven interviews. One interview was conducted in English by the researcher. The written responses were primarily in English, with one exception, which was translated by the interpreter.

Interpretation introduced methodological considerations. Interpretation is not a direct representation of the interviewee's words but a translation, and thus partially an interpretation. This required the researcher to exercise extra care in understanding and analyzing responses. Clarity during interviews was essential, with questions sometimes repeated to ensure accuracy. Follow-ups with participants were conducted to clarify ambiguities, and participants were asked to review parts of the article to confirm accurate understanding.

Interview durations ranged from 1.5 h, in the shortest case, to over 2 h in several instances (specific durations are detailed in Appendix 2). All interviews were transcribed verbatim.

### Inductive thematic coding

A thematic coding process based on inductive logic (Elo and Kyngäs, 2008; Thomas, 2006) was employed, as no prior research or deductive theory on military chaplaincy during Ukraine's war exists. The qualitative analysis software *Atlas.ti* was used to systematically manage and group codes into code families.

The analysis followed two main steps.

1) Open inductive coding: The transcriptions were systematically analyzed, and all noteworthy observations were marked and labeled. For example, if a participant mentioned, “prayer is an important preparation before combat,” this segment was coded as *prayer important before combat*. Similarly, if a participant stated, “confidentiality is an important release valve for military personnel,” this was coded as *confidentiality important release valve*. This initial phase generated 454 unique codes, many of which overlapped among participants.
2) Thematic organization into code families: To present the codes meaningfully, they were grouped into overarching themes known as code families in *Atlas.ti*. This is a more abstract form of coding, where the inductive approach moved toward generalization and deduction. A key principle in the process was to present the analysis findings in a coherent way, focusing on what military chaplains do in war and the lessons learned. Not all individual codes were automatically reflected in a code family; however, the associations of a code should lead to a code family, and vice versa. While some individual nuances were lost in the transition from specific codes to general themes, this process provided structure and clarity. This analysis resulted in the identification of 15 code families (see Appendix 3).

This article specifically draws on material from code family 6, which focuses on “Morality, ethics, and character formation in war,” and code family 14, which examines “Wisdom about the implications of war on soldiers, society, theology, Bible usage, etc.”

In the subsequent analysis, concepts and theory have been used to cross-fertilize the thematic coding. This is referred to as an abductive approach and aims to deepen the analysis (Vila-Henninger et al., 2024). It should be noted that *I* and *Me* are analytical concepts, with *Me* being the one that can primarily be approached empirically in research. Therefore, the focus of the analysis is on *Me*, while *I* remains an inseparable part of a person and, on a theoretical level, is entirely necessary.

Clear identity markers (e.g., names, ranks, geographical locations, specific events, and situations) have been omitted or blurred to make it more difficult to trace them back in the presentation of the analysis. Participants are referred to as, for example, MC 2 (Orthodox), and so on.

MC 9 has been given particular prominence in this article because his extensively articulated experience needed to be presented in more detail. His case was therefore deliberately not used extensively in previously published articles (Grimell, 2025a,b). However, his experiences are not unique, but correspond with many other experienced MCs who are also, of course, included in this article, although these accounts have been more extensively featured in earlier publications.

## Analysis

### Upholding and protecting morality, ethics, character, and the human aspect in war

MC 9 (Greek-Catholic) was a highly war-experienced chaplain who had voluntarily served as a frontline MC since the war began in 2014. He was one of the study participants with extensive and long-standing experience in military chaplaincy, dating back well before 2014. MC 9 shared that a key part of his work at positions along the frontline, as well as behind it, revolved around moral education, character, and attention with military personnel. This moral education and approach had a general character and was not dependent on any particular faith.

MC 9 (Greek-Catholic) recounted:

In the fields, I was always walking from one position to another position, and whenever I was there, or in a village or another village, or another position in the frontline, I was creating my own military ethos program. These included lectures that could be useful no matter the faith or denomination, talks with lessons on moral issues, moral value orientations, or shaping character to deal with combat stress or related challenges. This was my priority.

An important reason for this focus was the war's erosion of norms and rules, including the gradual value shifts that occur during a full-scale war like this. According to MC 9, the focus on moral education—*shaping moral character*—was as much about pastoral care as it was about nurturing the moral principles of the cultural *Me*. In the context of war, the work on moral principles and character was to be understood as pastoral care adapted to prevent or counteract the dissolution of social order and the unraveling of character, or the socially constructed and shaped *Me*, that follows war.

MC 9 (Greek-Catholic) explained:

One of the things that makes it difficult for people from the West to understand pastoral care [in war] is that it is still organized and structured for peaceful times in your countries, and that's exactly how civilization operates. There are a set of norms that regulate relationships between people. For example, you can schedule interviews between 8 a.m. and 4 p.m. But you can't do that in a war zone. War is about chaos—that's why norms and regulations don't function or protect people as they should. This is precisely why my number one priority was to recreate those norms and certain types of communication within the minds of the military personnel I spoke with. Otherwise, the psychological health and mental wellbeing of soldiers are at great risk when they lose any sense of order. Recreating that order involved articulating norms, discussing moral principles, and appealing to texts or prayers. My primary focus was the soldier's mind—that was what I was serving.

The cultural tools and resources that MC 9 used in his moral pastoral care approach to recreate and nurture a socially constructed *Me* were drawn from the Greek Catholic tradition to which he belonged as both a priest and an MC. This tradition encompasses theology, ethics, moral principles, pastoral wisdom, biblical narratives, rituals, prayers, sacraments, religious aids such as rosaries, sacred objects, medallions of the Blessed Virgin and the Angel Michael, prayer books, and more. It represents a timeless cultural knowledge that, through generations and in previous European wars, aims to uphold a Christian moral-ethical compass. This moral *Me* provides guidance to individuals in their relationships with themselves, others, right and wrong, and good and evil during war. Pastoral work provided military personnel with approaches and rituals for healing that went beyond and complemented military medicine (Illich, 1977).

The moral *Me* belongs in a social everyday context, which provides the *Me* with a certain sense of coherence—SOC, as Antonovsky (1987) describes—which war more or less brutally and profoundly disrupts. The concepts of comprehensibility, manageability, and meaningfulness become extremely strained by the war's dissolution of the everyday life's sustaining norms, values, and behaviors. Precisely because war uproots military personnel from their social contexts and distances them from these familiar surroundings, there arises a need to focus on their sense of coherence and everyday sense of *Me* to sustain morality, ethics, purpose, motivation, resilience, and the endurance of the human soul.

Most Western societies, regardless of secularization, can be said to have such an implicit moral-ethical *Me* or compass deeply embedded in the culture that underpins society. Societies seek to teach and instill such a moral *Me* in their citizens through socialization (Mead, 1934/2015). This *Me* shapes human character, organized around values and norms vital to society. However, war threatens to entirely dissolve these norms and rules encapsulated in the socio-cultural everyday Me, that is, to dissolve the socio-cultural *Me* carved out in a society shaped by peace, law, justice, ethics, and order. As such, the war poses acute stress to the part of the human soul here conceptualized as *Me*.

### Defending the humanity of military personnel who have lost their socio-cultural everyday reality in the service of war and killing

Although a stark departure from a peaceful society, the killing of other combatants is a natural part of war (French, 2005; Goldstein, 2001; Strachan, 2006; Verrips, 2006; Wilson, 2008). It is morally expected and required of a military *Me*. Yet even this is governed by the laws and conventions of war in order to maintain a kind of ethics and order in war. This not only concerns creating conditions for comprehensibility, manageability, and meaningfulness, but also aims to harmonize a military *Me* with a socio-cultural (civilian) *Me* to a greater extent within the human soul. An important way to maintain this is to nurture the integrity of a value-based view of humanity in a situation that threatens to completely dissolve morality and ethics, which, in turn, threatens to dissolve the socio-cultural *Me* that can be said to make people human and socio-cultural beings.

MC 9 (Greek Catholic) explained his approach to this process:

In the military, you have to do your job, and that involves fighting for your mission aims, your despairs, and your moral injuries, so to speak, but always according to certain principles and laws. This allows you to stay human in a situation that demands the collapse of humanity. I often say that war is the world beyond our measures. Of course, it is the greatest threat to humanity, especially this war.

It is important to follow laws and regulations because they protect the humanity from completely collapsing in war. This, in turn, would mean that the foundation for the *Me*, and thus the *Me* itself, depends on how well laws and regulations protect morality and ethics in a situation that seeks to collapse the socio-cultural conditions for comprehensibility, manageability, and meaningfulness. Deviations from the laws of war—abuse, brutality, torture, killing of civilians, bombing of civilian facilities and infrastructure, and so on—risk entirely dismantling the moral and ethical framework intended to organize human relationships in a wartime context. The witnessing and experiences of death, killing, and the ever-present threat of not seeing the next day, combined with the growing distance from everyday life and the everyday *Mes* that military personnel lived and upheld before the war, deeply affected them. The prolonged nature of the war further undermined the morality, values, and social identities learned in peacetime society. This erosion of values can have deeply undesirable consequences for the health of military personnel, the discipline and morale of units, and, ultimately, strip military personnel of their human *Me*.

The participants in the study testified that the war deformed soldiers and veterans (Grimell, 2025b). Of course, there were positive aspects, such as camaraderie and fellowship. However, the stresses of war, combined with the dissolution of morality, ethics, and sense of coherence, could lead soldiers to become war animals. A war animal referred to someone who had lost their human qualities, including the capacity for rational behavior and thought (Grimell, 2025b). Another highly experienced participant, who, like MC 9, had been on the frontline since the war broke out in 2014, had been a volunteer MC for much longer. He spoke about how challenging it was to handle the moral issues, maintain humanity, and avoid becoming a war animal.

MC 6 (Orthodox) recounted:

It requires long conversations, and it's not certain that it helps. This is one of the absolute hardest things, because many put on a mask in front. And some go completely crazy, “the top comes off” (explains the interpreter in Swedish), their heads explode. And this is difficult. The situations can be very complicated. We try to observe early signs of this, both chaplains and psychologists. Often, it happens in the form of excesses related to alcohol consumption, alcohol and all that stuff, then the activities they come up with themselves can completely spiral out of control, but we try to observe such things early.

The expression *mask* resonates particularly well with Wertsch's (1991) concept of *the mask of the warrior*, where she problematized the functions of the warrior mask: secrecy, stoicism, and denial, as well as alcohol abuse to relieve pressure. A warrior needs a mask (or military identity) to fulfill the role of a soldier, but the mask also has a downside that can have negative implications for mental health.

In a similar way, another very experienced participant emphasized the conversational approach in combination with monitoring negative mood in order to pastor military personnel.

MC 10 (Greek-Catholic) explained:

I listen and support the conversation. I monitor negative mood manifestations, and if they occur, I try to take appropriate measures based on the situation.

For the MCs in the study, staying vigilant and actively working to combat or mitigate the erosion of morals and character was a vital responsibility. Both in the immediate operational environment and over the long term, such losses of humanity posed a significant challenge for a military society engaged in waging war, as well as for a civilian society tasked with caring for its veterans. For military personnel and veterans, it was about the support and care of the integrity of their human soul.

Another very experienced MC explained that his main focus gravitated toward supporting military personnel so that, instead, they would activate and practice seeing the beautiful and uplifting aspects of war. This was a kind of pastoral method against being completely consumed and losing oneself in the darkness and destruction of war. The pastoral approach aimed to build resilience and prevent them from being broken down, and in the worst case, becoming a war animal.

MC 5 (Orthodox) stated:

It is my main job to work on this; I do it all the time. In situations like these, I try to highlight for the soldiers in dialogue what is good, what is working, camaraderie, the sky is blue, that there are many things around them. One has to try to find and articulate these things, even though it can be difficult.

MC 9 also applied a similar approach to counteract the destructive and degrading effects of war on the human being. The ability to seek and find meaning in a dynamic and changing situation, to identify a sense of coherence regardless of what was happening, was considered very important to protect and maintain one's human capacity and resilience.

MC 9 (Greek-Catholic) recounted:

Beauty is the most important thing. Once you are part of such a destructive force for such a long period of time, you don't know when something beautiful will appear around you—something that speaks to your heart rather than your mind. In such situations, you can easily lose yourself and cause irreparable damage to your humanity. To me, this is an important behavioral state and sense: to have a taste of life, to see a sense in what is going on, and to sustain mental wellbeing, especially in the combat zone. I usually offer some exercises to the military to increase their resilience by enhancing their taste for life, their appreciation of death, and how to find meaning in what is happening. For many people, faith does this: it provides avenues to reconstruct reality by finding meaning in whatever is happening around you and in the routine.

Among the MCs, there was a clear emphasis on preserving the individual humanity of military personnel and upholding broader human ethics in the context of war, where human dignity was often severely challenged.

MC 2 (Orthodox) recounted:

It is very important to maintain the human side of oneself, even when external circumstances are very challenging.

This applied not only to individual soldiers in relation to their emerging ethical mindset, actions, and character, but also to military commanders. The MCs described the importance of making commanders aware of the ethical dimension in their decisions, how to act toward the enemy, and the need to resist giving in and losing the good ideals and ethical norms (Grimell, 2025b).

### Intense emotions and the drive for revenge in relation to prisoners of war

The erosion of morals and character, and its implications, can be particularly challenging when the enemy becomes captive. Regardless of whether the Russian soldiers taken prisoner were personally responsible for the killing of battle buddies, colleagues in other units, or attacks on civilians, they became a concrete symbol of the killing, the Russian aggression against Ukraine, and potential violations of the laws of war committed during the conflict. The feelings and desire for revenge could be palpable in such situations. However, for several reasons, maintaining a strong moral *Me* and character in such situations was crucial.

MC 4 (Orthodox) recounted:

Something that is also a major issue, and that we talk about a great deal, is how soldiers should relate to their adversary, to the enemy, and when they capture them. That is, the people who have tried to take their lives, those of their children and families, and so on—how they should approach that and them. We've worked on this quite a bit. I explain the consequences of revenge and how it can affect a person. But also that it is not to Ukraine's advantage to take the life of a prisoner, because a prisoner can be valuable. I also highlight the importance of, and explain to the soldiers, that destructive actions can make things dangerous, especially for your comrades when it comes to the handling of prisoners. As a priest, I don't talk about bad people but rather bad actions. It is the bad actions I address as a priest.

These types of MC experiences from the war in Ukraine highlight how essential it is to prepare for and continuously work on the handling of prisoners of war from moral, ethical, and character perspectives. This is especially urgent when the laws and rules of war are violated, battle buddies, families and close relatives have died, been injured, or subjected to bombings, artillery, and drones, combined with rapid media dissemination and disinformation.

This is not only about how moral transgressions can lead to moral injuries that are difficult to live with in the aftermath of war (Shay, 2002, 2003; Litz et al., 2009; Koenig et al., 2023). It is also connected to critical intelligence information, the West's perception of Ukraine, and the integrity of the Armed Forces of Ukraine. Ultimately, this is about protecting the moral character and integrity of the individual, the armed forces, and the nation as a whole.

### Practicing pastoral care through narratives that create a sense of coherence

Many participants used a narrative approach in the pastoral care they practiced and the moral education and character-building efforts they undertook. This narrative approach could draw its material from various biblical sources. It was a way to connect a soldier to a Christian tradition and culture with sacred undertones, creating a sense of coherence, purpose, and meaning, as well as providing spiritual comfort and solace for soldiers facing various challenges. Or simply as a confirming biblical narrative for an already focused soldier or group in preparation for their mission.

MC 12 (Protestant) explained that he used the following Bible stories when practicing pastoral care for combat military personnel on the front lines in various situations:

Gospel stories of faith in Jesus: there are two different centurions who saw and believed in Jesus; there is Cornelius the centurion in the Book of Acts; there is David the warrior and his many psalms, especially Psalm 91; there is Peter walking on water and then drowning in the storm, with Christ reaching out a helping hand in response to his urgent prayer; there are the apostles in dire straits in the storm, and Jesus asking them, “Where is your faith?”; there is Paul in Romans 13 talking about “the minister of God,” that “he beareth not the sword in vain: for he is the minister of God, a revenger to [execute] wrath upon him that doeth evil.” Finally, there is the Prodigal Son returning to the Father.

MC 10 (Greek-Catholic) stated:

The most authoritative source of wisdom is the Holy Scriptures.

These authoritative Christian cultural scripts offered both relevant themes and biblical characters with whom military personnel and veterans could identify. The opportunities provided by the religious frameworks could be employed to mitigate struggle, hardship, suffering, and pain, and nurture healing. Religion as culture can provide frameworks and clues on how pain and suffering should be endured through stories, symbols, and examples. There is actually research on biblical combat veterans that has not been used in this context but could be highly relevant for a type of pastoral care that is open to employing such an approach (Grimell, 2022).

Another very important narrative element in the pastoral care practiced by the participants, and conveyed to military personnel, was, of course, the broader narrative of their role as protectors of the country, civilians, and the next generation. Such a grand narrative served several functions. It was about creating a strong moral narrative for the role of a soldier, which included sacrificing one's life for something greater than oneself (the independence of the country, the safety of civilians, and the next generation being raised in a democratic and independent society). Thus, the narrative aimed to instill the will and courage to sacrifice oneself for something larger—that is, a willingness to fight and die.

This narrative approach also emphasized standing behind military personnel, showing solidarity, and spiritually strengthening them. At the same time, the narrative raised the religious, moral, and legal legitimacy of using violence. The narrative was also employed to pastorally care for and assist those who were sad, scared, and filled with anxiety in their role as combatants (see also Grimell, 2025b).

MC 6 (Orthodox) recounted, with his 10 years of experience in frontline military chaplaincy:

Soldiers are worried about the future and how this will end. Simply about Ukraine's future. But it also happens, although not very often, that soldiers question the use of weapons and what right they have to take the lives of others. In those cases, I try to explain their role as protectors, which might make it easier for them to cope with it.

MC 8 (Greek-Catholic), with equally long experience, explained:

In the situation Ukraine is in right now, where we are so heavily attacked by Russia, the church's content has changed so that songs and prayers are primarily dedicated to strengthening the armed forces. The most important task for the soldiers is to act as protectors of Ukrainian society, civilians, the territory, and, indeed, the next generation.

From a theological perspective, Ukraine's situation also aligns well with the theory of a just war, which encompasses two general clusters of principles – *Jus ad Bellum* (the right to go to war) and *Jus in Bello* (justice in war) (Holmes, 2005). The first stipulates that the following criteria must be met for a war to be initiated for a just purpose: (1) a just cause (e.g., a country is attacked by an aggressive opponent and must defend itself), (2) legitimate authority, (3) right intention, (4) proportionality (the war must not cause more harm than good), (5) assurance that it is a last resort, and (6) a purpose to achieve peace. *Jus in Bello*, on the other hand, aims to limit the scope and conduct of war once it has started. For a war to be conducted justly, two criteria must be met: (1) adherence to the principle of proportionality (relating to weapons and the extent to which they may be used) and (2) discrimination between combatants and non-combatants.

Since these principles can be said to be fulfilled in Ukraine's case, and because the Western world, which provides weapons to Ukraine, also tends to limit the use of certain potent weapons (at least so far), an especially important control mechanism for *Jus in Bello* exists. If the principles of just war had not been fulfilled, it might not have been quite as straightforward to use both biblical sources and the broader narrative to strengthen and legitimize the sacrifice of oneself and the killing of other combatants in the name of independence and freedom. This, in turn, would potentially have made military personnel more vulnerable to what is referred to as spiritual injury (Berg, 2011) and moral injury (Shay, 2002, 2003; Litz et al., 2009; Koenig et al., 2023).

### Recreating a sense of coherence that provides *Me* with meaning and *I* in a moral context

To activate, uphold, and protect the humanity of military personnel and others whose existence had been shattered by the war was crucial to the MCs. As already showcased, the protection of humanity encompassed, among other things, moral and ethical education, the shaping of moral character, and exercises and reflection to identify the uplifting, beautiful, and meaningful in a destructive, dark, and changing situation. Additionally, this also involved recreating reality, establishing a sense of coherence (Antonovsky, 1987), and reinstating a value-based meaning and direction in life using the cultural tools at his disposal.

MC 9 (Greek-Catholic) explained:

To recreate reality—this is so important to me. This is a priority task for a chaplain: to be there with a story, with a prayer, with a Bible story, with whatever, with ethos, but to provide certain types of reality, to reconstruct reality with some moral orientations, with something value based. But once again, protecting humanity.

The *Me* that society provides to an individual encompasses the morals, values, meanings, and practices that constitute a person within a social context (Mead, 1934/2015). A war and the traumatic events that follow risk dissolving the *Me* that forms the social human being. If this *Me* erodes or even dissolves entirely, ceasing to hold any relevance for a person, it becomes difficult to exist, to orient oneself in life, or to live. This is not merely an emotional trauma but something much deeper. The dissolution of a *Me* equates to an existential erosion of life, threatening life itself and the will to live (Tillich, 1952/2014).

This also means that the *I* is left entirely unanchored, without a cultural context that provides the *I* with a surface to hold onto or push against morally, meaningfully, and in terms of actions. The uniqueness of an *I* is tied to context and culture, which are embodied by one or more *Mes*. If the meaning of such a cultural structure in the self dissolves, the question of the *I*'s originality and uniqueness is at stake. *I* is only unique and original in relation to culture and context; if this is dissolved, *I* is deprived of its existential being in life (Tillich, 1952/2014). The mutual dependence between *I* and *Me* for the human soul cannot be overstated, as one needs the other and vice versa.

The participants in the study were acutely aware of the importance of monitoring and counteracting the dissolution of morals, norms, rules, and character using the religious and cultural toolkits at their disposal. Thus, the Ukrainian MCs could be described as *the defenders of the human soul* among military personnel, veterans, families, and others they supported within the scope of their mission.

## Discussion

There are several takeaways from the analysis that need to be discussed further in this concluding part of the article.

### The disintegration of everyday reality in war goes far beyond medicine's solutions

War has always proven to be a brutal social reality that dismantles the social order and structure of peaceful societies, particularly among military personnel (French, 2005; Goldstein, 2001; Strachan, 2006; Verrips, 2006; Wilson, 2008). Values, norms, meaning, and practices change radically during wartime; previously held beliefs no longer apply to either soldiers or civilians. This challenges and erodes a socio-cultural *Me*. The self-transformation to war and the struggle to find a way back after war is a turbulent and taxing process for both society and the individual combatant (Lifton, 1992; Shay, 2002, 2003; Tick, 2005).

Military personnel must also cope with the constant threat of death, killing other combatants, witnessing and losing battle buddies, experiencing abuse and war crimes, losing the familiarities of everyday life, and being separated from loved ones (Grimell, 2025b), among other challenges. All these stresses and the suffering that follows affect the mental health and wellbeing of military personnel and veterans (Figley and Nash, 2015; Lifton, 1992; Shay, 2002, 2003; Tick, 2005). However, the negative existential, ethical/moral, social, and relational effects of war on health cannot be confined to a purely medical perspective (Koenig et al., 2023; Shay, 2002, 2003). On the contrary, the disintegration of the peaceful society's everyday reality in war—which is reflected in the disintegration of a socio-cultural *Me*—extends far beyond the solutions medicine offers for health problems. This type of challenge may perhaps be best approached by a broad existential cluster that encompasses issues tailored to the social, cultural, ethical, moral, *Me*, and what it means to be human.

MCs—priests, pastors, imams, rabbis, religious and humanist representatives—represent the cultures on which societies are built; in other words, what can be called our human, culturally and socially constructed reality (Grimell, 2025a). Indeed, MCs may take a more traditional, specific approach in some aspects of their profession, such as administering sacraments and leading worship. But as the analysis clearly illustrates, the participants in this study expressed that a significant part of their work involved upholding and protecting morality, ethics, character, and the human aspect among military personnel in war. War dismantled the values, norms, and rules that made up the soldiers' human dimensions. This dehumanizing process was a direct threat to mental health. MCs described themselves as protectors of humanity in war, and this approach to military personnel was general, tailored to individuals, and context-specific situations.

In light of the conceptualization presented earlier, I describe them as the defenders of the human soul. With the support of their cultural religious traditions and the toolkit at their disposal—including biblical stories and the broader narrative as protectors of the country—they helped military personnel activate, uphold, and protect the human aspects, or everyday Me, throughout the war. When military personnel lost their sense of coherence (Antonovsky, 1987), MCs helped recreate a sense of coherence that provided meaning and moral context. This was a hard and continuous task that had no checklists or evidence-based procedures (Grimell, 2025b). Despite their extensive experience in wartime military chaplaincy, they needed to improvise and meet each soldier from their unique identity, perspective, and situation. An important aspect that facilitated such a pastoral approach and conversations was that MCs had confidentiality, unlike medical staff, who had a duty to report if anything affected a soldier's mental fitness for service (Grimell, 2025b). In line with Illich's (1977) argument, it can be said that the MCs' pastoral approach to military personnel enhanced their natural capacity to cope with existential mental health challenges during the war.

### The role of poetic interpretation in supporting the human soul

A critical perspective on modern medicine, illness, and suffering—often employed within medical sociology, medical anthropology, and medical history—was put forward by Illich in his influential and thought-provoking book *Limits to Medicine: Medical Nemesis – The Expropriation of Health* (1977). Illich argued that modern medicine, despite its achievements, could become destructive by over-medicalizing life and undermining people's natural capacities to cope with illness and suffering. He contended that medical institutions frequently created new problems, including iatrogenic illnesses (caused by doctors' medical interventions), and fostered dependence on professional care at the expense of individual autonomy and alternative cultural understandings. The reliance on medical expertise and interventions during times of suffering can overshadow the search for a *poetic interpretation* of one's predicament or the admiration of those who have learned to endure suffering. Other cultural approaches to addressing suffering were gradually displaced by an overly dominant medical paradigm.

Illich took his critique of modern medicine (also see Conrad, 2005; Conrad et al., 2010; Bradby, 2012) and pharmacology's growing dominance, power, and vast economic interests quite far. A key aspect of his thought-provoking argument about medicine's position of power is the erosion of people's—and indeed entire socio-cultural systems'—capacity and empowerment in managing suffering (Illich, 1977, also see Kleinman, 2014). As soon as suffering arose, expert knowledge was and is primarily sought within medicine.

The medical dominance interacted with a socio-cultural process commonly referred to as secularization (Casanova, 1992, 1994). In secular Western societies, where medicine holds a dominant position, only a small fraction of the society's former ritual healing roles remain. This is highly unfortunate, as, for instance, major religions reinforce resignation to misfortune and provide a rationale, a style, and a communal setting in which suffering can be transformed into a poetic and dignified experience (Illich, 1977; cf. Higgins, 2020, 2024).

The opportunities provided by the acceptance of the soul's suffering are interpreted differently across major religious traditions: as karma accumulated through past incarnations, as an invitation to Islam—the surrender to God—or as an opportunity for closer association with the savior on the Cross. Religion is culture, and throughout history, religions have always provided examples of how pain and suffering of the soul should be endured—whether through the Buddha, Christ, the saint, the warrior, or the victim (Illich, 1977). Religions stimulate personal responsibility for healing, summon consolation that is sometimes pompous yet sometimes effective, provide saints as role models, and typically offer a framework for the practice of folk medicine. However, the medicalization of suffering and pain has led to a hypertrophy of just one of these cultural modes while accelerating the decline of the others.

From Illich's perspective, the need for alternative cultural understandings of human suffering, beyond medical practices and discourses, highlights the important role and function of military chaplaincy services (Carey et al., 2016; Grimell, 2023; Koenig et al., 2023) and chaplains in a medical context (Bradby, 2016). In particular, within a military medical context, the need for alternative cultural perspectives—represented by MCs—has gained increasing relevance in light of major Western joint operations in the early 21st century (Grimell, 2024).

War attacks and dissolves prevailing norms, values, and cultural foundations, exposing military personnel to severe existential and mental strain, leading to deteriorated mental health, suffering, and pain (Figley and Nash, 2015; Grimell, 2025a,b; Litz et al., 2009; Shay, 2002, 2003). The dissolution of culture's norms, values, and character is the reason why the human soul is significant and an important concept for understanding MCs' work in war. They work just as consciously to combat and prevent the dissolution of the cultural human part of the soul (*Me*), as they do to support and pastor the unique and original part of the soul (*I*). The horrors of war are equally destructive to both *Me* and *I*. MCs are the defenders of the human soul, a concept that deepens the understanding of their work, which goes far beyond merely practicing pastoral care and performing rituals (Grimell, 2025b).

War is existential—one of life and death—both on an individual level and a societal level. People die, and the victor defines the social system, ideology, and culture that will prevail once the war ends. A full-scale war is therefore profoundly existential, not pathological or medical. If the implications of war on humans are addressed solely from a military medical perspective, then, as Illich (1977) put it, only a small fraction of a society's traditional healing roles is being utilized.

The cultural knowledge and wisdom of MCs can be far more pertinent and advanced than, for example, a medical clinical understanding of the existential, spiritual, and moral experiences, including suffering, that military personnel and veterans encounter during and after war (Bobrow et al., 2013; Brock and Lettini, 2013; Koenig et al., 2023; Smith-MacDonald et al., 2018; Wortmann et al., 2017).

### Advancing the understanding of war-related moral and spiritual injuries

Finally, war provides a particularly fertile ground for existential struggles and moral issues (Figley and Nash, 2014). This is something that has relatively recently come to be referred to as moral injury (Shay, 2002, 2003) and spiritual injury (Berg, 2011). This complex of problems has been understood and addressed to varying degrees within Western healthcare, although it may look quite different in different countries. The cultural knowledge and wisdom of such issues among wartime MCs can be far more pertinent and advanced than, for example, a medical clinical understanding of the existential, spiritual, and moral experiences—including suffering—that military personnel and veterans encounter during and after war (Berg, 2011; Bobrow et al., 2013; Brock and Lettini, 2013; Koenig et al., 2023; Smith-MacDonald et al., 2018; Wortmann et al., 2017).

Moral and spiritual conflicts and injuries are linked to a domain of character (Atuel et al., 2021; Shay, 2002, 2003) and to one or more *Me's* that have clashed, been violated, or broken (Grimell, 2023). The MCs' work with ethics, morality, character, stories, and narratives explicitly and implicitly aimed to prevent and avoid the deep-seated moral and spiritual injuries from arising as much as possible. A particularly difficult and conflict-laden area was the family domain. At the time of the interviews, the full-scale war had been ongoing for 2.5 years, which meant significant strain in maintaining a *Me* that originated from and emanated within a family context. The theme of family, conflict, and frustration was something that participants in the study experienced as a challenging issue that could not be resolved as long as the war continued. How extensive the moral aspect of this struggle will be once the war eventually ends remains to be seen, as well as how recovery from the suffering it has already caused and may continue to cause can be supported.

### Limitations and future research

When it comes to the conceptualization of the human soul and its application in the analysis, *Me* (Mead, 1934/2015) has been used as a generic term for the culturally and socially constructed part of the soul in the self. A person obviously has many *Mes*; different civilian *Mes* or social identities (e.g., as a man, married man, father, son, football player, etc.) and, of course, a military *Me*. The point of the analysis was not to illustrate the interaction between different *Mes*, as the methodology does not allow for this. Rather, the idea was to relate the strong focus on protecting morality, ethics, character, and the human aspects—that is, the socio-culturally constructed Me (Mead, 1934/2015)—to the human soul. *Me* is something that can be empirically derived from socio-cultural identity claims. *I*, on the other hand, represents the spiritual aspect of a person, *I* operates on the theoretical level, and cannot be empirically captured, but may be described by a subject. The soul remains an inseparable part of the human (socio-cultural *Me*) of a person.

A central observation from this study, drawn from the participants' reflections on themselves and others, is that exceptions are an inevitable part of wartime. These exceptions must be understood within the broader context of large-scale war, the professionalization and institutionalization of military chaplaincy, and the inherent constraints of qualitative research methods (Grimell, 2025a,b).

The purpose of a qualitative study is to deepen our understanding of people's experiences and to explore the phenomenon being examined—in this case, the roles, responsibilities, and insights gained by MCs during wartime. From this perspective, the study can be deemed successful, as it involved highly experienced interviewees. However, there were language barriers that were addressed through the use of an interpreter, which added a layer of mediation between the firsthand experience and its translation. Given that this is a qualitative study, readers should exercise caution when making generalizations. The experiences shared by these participants may not reflect the experiences of all MCs. A recurring conclusion from the interviews was that exceptions are a constant feature in wartime.

A field-based study could also offer deeper insights into the interactions between MCs and churches or religious communities. Expanding the research to include interviews with priests, military commanders, veterans, and families would further enrich our understanding—especially from their perspective—of the importance and role of military chaplaincy during conflict. Such an expanded study would also highlight the urgent need to address the issues faced by veterans, a responsibility that churches, religious communities, and MCs would be well-placed to focus on.

## Data Availability

The original contributions presented in the study are included in the article/Supplementary material, further inquiries can be directed to the corresponding author.

## Footnotes

^1^When it comes to, for example, the number of MCs in Ukraine, how many have died or been injured in the war, the researcher has taken the following stance: No figures are mentioned. This is partly because (a) war involves war propaganda, (b) partly because Ukraine does not release figures on casualties, and (c) partly because intelligence agencies are piecing together information. Various types of public figures circulate on the internet regarding all these matters, but the researcher has chosen not to publish internal figures mentioned during the interviews, as it is not in Ukraine's interest to do so.
