# Supplementary material for: Understanding Ukrainian military chaplains as defenders of the human soul
Source: Front Sociol. 2025 Mar 12;10:1559023. doi: 10.3389/fsoc.2025.1559023 (PMC11936900; doi:10.3389/fsoc.2025.1559023)
Supplement: Supplementary file 1 [file Data_Sheet_1.docx]

Appendix 1

Interview guide for the study on MCs in War

**(A) Background information**

1. Could you start by telling us a little about yourself, such as who you are, how old you are and where you come from?

2. Would you like to tell us a little about your background as a priest and chaplain?

3. How did you get into the role of military chaplain?

4. How long have you been a military chaplain?

5. Where have you worked as a military chaplain?

**(B) Roles and tasks**

6. What are the tasks of a military chaplain?

7. What would you say are the most important and central tasks you perform as a military chaplain?

8. Do you perform other tasks, such as military ones, that are outside the scope of spiritual/pastoral care?

9. Do you carry weapons as a military chaplain?

10. Who do you obey as a military chaplain?

11. What does a typical day look like for you as a military chaplain?

12. What does a typical week look like for you as a chaplain?

13. Has military spiritual care changed or developed since the war - If yes, please describe in what ways?

14. Does military spiritual care become more urgent in war – If so, describe in what ways?

**(C) Moral, Spiritual and Existential Challenges in War**

15. Could you tell us a little about the issues you encounter with those you support and counsel?

16. What are the common reasons or themes of those who want to talk to you?

17. What moral questions do you encounter in the minds of those you support and counsel?

18. What spiritual questions do you encounter in the minds of those you support and counsel?

19. Are there other types of existential questions, i.e. questions about life, that you encounter in the minds of those you care for?

20. How do you usually approach these different issues?

21. What rituals, such as blessing, intercession, communion, worship, funeral or the like, do you think are important?

22. In what situations are these rituals important?

23. Is there a particular type of story, wisdom, or other item from your religious tradition that is particularly useful when you practice pastoral counseling?

**(D) Important for maintaining the ability to exercise military spiritual/pastoral care in war**

24. Have you received any special training for your assignment as a military chaplain?

25. If no, please describe education, skills or competencies that you would have liked to have received before?

26. What is important in order to be a good chaplain in wartime?

27. Have you developed or changed as a military chaplain in wartime—if so, in what ways?

28. In your opinion, what is good military care and counselling in wartime?

29. In what ways do you feel that spiritual/pastoral care helps those you meet?

30. Are you usually able to see or otherwise perceive the "effects" of spiritual care? If yes, please describe?

31. How have you maintained the ability to minister/conduct military spiritual care in wartime? What is important to maintain the ability over time?

**(E) Conclusion**

33. Now the interview is over, is there anything you would like to come back to in the interview or add?

34. Is there any question I should have asked in order to better understand military spiritual care and chaplaincy in wartime, and if so, what one?
