# Supplementary material for: Understanding Ukrainian military chaplains as defenders of the human soul
Source: Front Sociol. 2025 Mar 12;10:1559023. doi: 10.3389/fsoc.2025.1559023 (PMC11936900; doi:10.3389/fsoc.2025.1559023)
Supplement: Supplementary file 2 [file Data_Sheet_2.docx]

**Appendix 2**

Interview Time (hours, minutes, seconds)

Interview MC 1 = 1.31.30

Written account MC 2

Interview MC 3 = 2.09.36

Interview MC 4 = 2.07.41

Interview MC 5 = 1.35.42

Interview MC 6 = 1.49.24

Interview MC 7 = 1.36.53

Interview MC 8 = 1.53.21

Interview MC 9 = 1.43.21

Written account MC 10

Written account MC 11

Written account MC 12
