# Supplementary material for: Understanding Ukrainian military chaplains as defenders of the human soul
Source: Front Sociol. 2025 Mar 12;10:1559023. doi: 10.3389/fsoc.2025.1559023 (PMC11936900; doi:10.3389/fsoc.2025.1559023)
Supplement: Supplementary file 3 [file Data_Sheet_3.docx]

**Appendix 3**

| **Code families: Thematic Coding of Interview Data** |
| --- |
| 1. Background of the participants |
| 1. The development of military chaplaincy in Ukraine (institutionalization and professionalization) |
| 1. Main duties today |
| 1. What the MC does when the unit is not engaged in combat |
| 1. What the MC does when the unit is engaged in combat |
| 1. Work involving morality, ethics, and character formation in war |
| 1. Availability and presence for military personnel |
| 1. The role of confidentiality in war |
| 1. Perceived spiritual/religious needs during war |
| 1. Questions and issues that military personnel bring to the MC in wartime |
| 1. How the MC is affected by the war |
| 1. How MCs maintain spiritual resilience during war |
| 1. Qualities of a good MC in war |
| 1. Wisdom about the implications of war on soldiers, society, theology, Bible usage, etc. |
| 1. The importance of church structures outside of the armed forces during war |
